# Severe acute respiratory Syndrome-Coronavirus-2: Can it be detected in the retina?

**DOI:** 10.1371/journal.pone.0251682

**Published:** 2021-05-13

**Authors:** Tarek Bayyoud, Angelika Iftner, Thomas Iftner, Karl Ulrich Bartz-Schmidt, Focke Ziemssen, Hans Bösmüller, Falko Fend, Jens Martin Rohrbach, Marius Ueffing, Michael Schindler, Sebastian Thaler

**Affiliations:** 1 Department of Ophthalmology, University Hospital Tübingen, Tübingen, Germany; 2 Institute for Medical Virology and Epidemiology of Viral Diseases, University Hospital Tübingen, Tübingen, Germany; 3 Institute of Pathology and Neuropathology, University Hospital Tübingen, Tübingen, Germany; 4 Institute for Ophthalmic Research, University of Tübingen, Tübingen, Germany; University of Florida, UNITED STATES

## Abstract

**Background/Objectives:**

The systemic organ involvement of SARS-CoV-2 needs to be thoroughly investigated including the possibility of an ocular reservoir in humans. To examine retinal tissues and vitreous for histopathology and SARS-CoV-2 presence with regard to possible effects on the human retina and/ or vitreous. We performed histopathological analyses and quantitative (q)RT-PCR-testing for SARS-CoV-2 RNA on retinal tissues and vitreous of COVID-19 postmortem donors.

**Subjects/Methods:**

Included in this study were 10 eyes of 5 deceased COVID-19 patients. The diagnosis of SARS-CoV-2 infection was confirmed via pharyngeal swabs and broncho-alveolar fluids. The highest level of personal protective equipment (PPE) and measures was employed during fluid-tissue procurement and preparation. Histopathological examinations and qRT-PCR-testing were carried out for all retinal tissues and vitreous fluids.

**Results:**

The histopathological examinations revealed no signs of morphologically identifiable retinal inflammation or vessel occlusions based on hematoxylin and eosin stains. By qRT-PCRs, we detected no significant level of viral RNA in human retina and vitreous.

**Conclusions:**

In this study, no significant level of SARS-CoV-2-RNA was detected in the human retinal and vitreous fluid samples of deceased COVID-19 patients. Histopathological examinations confirmed no morphological sign of damage to retinal vasculature or tissues. Further studies are needed to confirm or refute the results.

## Introduction

The ongoing spread and dissemination of Severe Acute Respiratory Syndrome-Coronavirus-2 (SARS-CoV-2) into the community poses unprecedented challenges for the healthcare system. This includes healthcare management, patient guidance and surgery on potentially infectious, asymptomatic SARS-CoV-2 carriers. Bearing in mind the high volume of cases in ophthalmological practice and the physically close ophthalmologist to patient contact during eye examinations and interventions, precaution should be taken to reduce pathogen transmissions.

The different modes of spread and systemic organ involvement of SARS-CoV-2 need to be thoroughly investigated including the possibility of viral reservoirs in humans. The question if this novel member of the coronavirus family, possibly exhibiting similarities in viral transmission and/ or organ involvement to members of the flavivirus family, as described by Khaiboullina et al. [1] in 2019 and Kitagawa et al. [2] in 2018 for the Zika Virus and West Nile Virus, respectively, remains still unanswered. A proven similarity with certainty in viral spread in human tissues and/ or fluids of SARS-CoV-2 would not only be concerning but also quite novel for its family. As recently described by Marinho et al. [3], there might be retinal damage in the context of COVID-19, a finding the authors confirmed with a larger cohort of more than 150 patients [4]. Schnichels et al confirmed the presence of angiotensin-converting enzyme 2 (ACE2) and transmembrane serine protease 2 (TMPRSS2) in retinal tissues [5]. Thus, SARS-CoV-2 might potentially directly infect the human retina and cause tissue damage. It was further suggested that the virus is able to cause a follicular conjunctivitis [6] with the potential to invade further via fluid translocations. A most recent report by Casagrande et al. [7] states–although with not unequivocally test results–positive retinal biopsy samples of human retina.

Hence, it is important to carefully analyze vitreal and retinal tissue samples of COVID-19 deceased for the presence or absence of SARS-CoV-2 viral RNA. Accordingly, the objective of this study was to examine vitreoretinal involvement in COVID-19 postmortem donors based on detection of SARS-CoV-2 viral RNA and tissue damage analyses via hematoxylin and eosin stain. Moreover, as described by Barton et al. [8], we would like to share our experience regarding personal protective equipment (PPE) used and necessary measures taken during these tissue-/ fluid-procurements and preparations.

## Materials and methods

### Informed consent, approval of independent institutional review board

Written informed consent, adherence to the Declaration of Helsinki and approval of the independent Ethics Committee of the University of Tuebingen (institutional review board) was obtained prior to commencement of the study (241/2020BO2). All experiments were performed in accordance with the guidelines of the Corneal Bank of the Department of Ophthalmology of the University of Tuebingen and the governmental regulations.

### Tissue procurement

#### Specific guidelines, assessment of the environment for tissue procurement, and personal protective equipment

Guidelines for the enucleation team (two persons). To be checked prior to the enucleation of a COVID-19 postmortem donor:

The place of enucleation was defined as an area and/or room needing permission to access. The time spent at the location had to be documented. In addition, the place of enucleation was not allowed to be used by another person at time of tissue extraction. Any kind of aerosol and/ or turbulence had to be prevented. The necessary equipment had to be discarded after usage and/ or disinfected depending on the specific utensils used. To preclude any kind of self-harm PPE had to be used appropriately and operating procedures followed. This included surgical hand disinfection; gowns (overalls and apron); double gloves (as indicator system); and hood; face mask (FFP-3 level: 0.6μm/ 99% filtration); surgical instruments with tray; disposal of infectious wastes in a one-time lockable container; and of sharp utensils in a suitable, second container.

*Enucleation and preparation protocols*. A routine tissue procurement protocol for bio-banking was employed for the left globe of each donor. The respective right globe was kept naïve during the enucleation and preparation steps. The protocol employed was as previously published in detail and as to current guidelines at that time [9].

*Enucleation*. The enucleation was performed at the designated COVID-19 autopsy room of the Institute for Pathology and Neuro-pathology of the University Hospital of Tübingen. The average time of death to retrieval was 21 hours.

*Transport*. Transportation was done via a re-lockable, marked container (“COVID-19 donor tissue”); temperature was recorded and kept between 33.8°F to 50°F (+1 to +10°C) using cooling packs and a box avoiding contact to ice (Libero T1, Elpro, Switzerland); with direct preparation and further testing of donor tissue or storage at 42.8°F (6°C).

**Fig 1:**
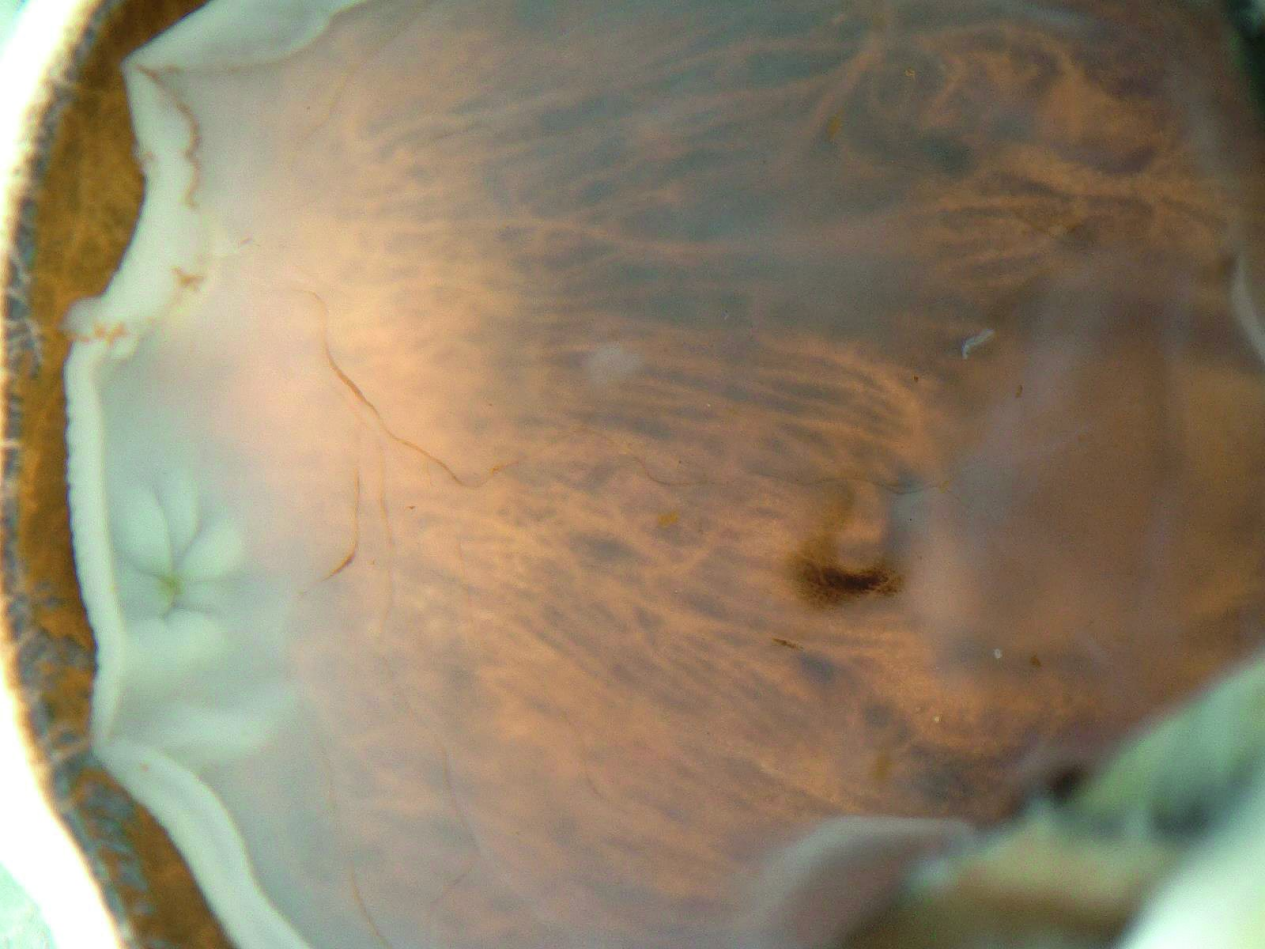
COVID-19 postmortem donor eye. Dissected donor eye depicts a detached retina, optic disc and retinal vessels prior to retinal dissection.

*Preparation*. The preparation was performed at a BSL2 laboratory (under a sterile workbench) of the Institute for Medical Virology of the University of Tübingen (Fig 1). The average time of death to preservation was 31 hours.

*RNA digestion and extraction*. We used the following kits according to the manufacturer’s instructions: RNeasy Kit, QiaCube, QiaSymphony DSP Virus/ Pathogen Kit (Qiagen, Hilden, Germany); RealStar SARS-CoV-2 RT-PCR Kit 1.0 (altona Diagnostics GmbH, Hamburg, Germany); LightMix® Modular SARS-CoV (COVID19) kit (TIB Molbiol Syntheselabor GmbH, Berlin, Germany); LightMix^®^ SarbecoV E-gene Kit (TIB MOLBIOL, 40-0776-96; TIB Molbiol Syntheselabor GmbH, Berlin, Germany) and Roche LightCycler^®^ Multiplex RNA Virus Master kit (Roche, 07083173001; Roche Molecular Systems, Inc., Pleasanton, USA). Key steps included: RNA extraction and quantitative Reverse Transcription-Polymerase Chain Reaction based on quality approved protocols with controls (S1 File). DNAse digest with RNAse-Free DNase Set (#79254), purification with RNeasy Mini Kit (Qiagen #74106) (S2 File). quantitative Reverse Transcription-Polymerase Chain Reaction (S3 File).

*Histopathology*. A histopathological macroscopic and microscopic examination using standard hematoxylin and eosin stains was performed on the extracted fluids and tissues.

*Patient characteristics and clinical aspects of COVID-19*. Patient characteristics, type of care and organ system involvement of the COVID-19 patients have been described earlier in detail (Bayyoud et al. Absence of Severe Acute Respiratory Syndrome-Coronavirus-2 RNA in Human Corneal Tissues. Cornea. 2021 Mar (Table 1 & 2)) [9]. Patient characteristics included past medical history, past drug history and time of hospitalization. Initial patient presentation was with unspecific symptoms and progression to a full picture of COVID-19 in all patients. Pharyngeal swabs and bronchoalveolar lavage fluid were tested positive for SARS-CoV-2-RNA by qRT-PCR. Coinfections with herpes simplex virus, cytomegalovirus, respiratory syncytial virus, parainfluenza, and influenza were excluded using qRT-PCR. The type of care included supportive, respiratory intubation and machine-assisted support including extracorporeal membrane oxygenation (ECMO) and continuous venovenous hemofiltration. Organ system involvement was extensive in all cases and included the respiratory, gastrointestinal and urogenital systems. A life-threatening organ dysfunction was diagnosed in all patients leading to the involvement of the gastrointestinal and genitourinary systems. A diagnosis of multi-organ dysfunction syndrome (MODS) was finally made in 3 patients. The respiratory and renal systems were involved in all patients leading to Acute Respiratory Distress Syndrome and kidney failure, respectively. Postmortem pulmonary tissue samples from COVID-19 deceased were tested for SARS-CoV-2 RNA via RT-PCR. All tested samples had positive SARS-CoV-2 results.

## Results and discussion

We report the absence of SARS-CoV-2 RNA in the human retina and vitreous obtained from COVID-19 cadaveric postmortem donors. SARS-CoV-2 RNA was not detectable in retinal tissues and vitreous fluids using qRT-PCR. All tissue and fluid samples tested negative for SARS-CoV-2 viral RNA amplifying the viral S and E genes (**Table 1**). Histopathological examinations revealed no signs of ischemic sequelae or lytic cellular lesions. With the exception of epi-retinal membranes, the microscopic and macroscopic histopathological examinations performed confirmed in all globes normal extra- and intra-ocular morphology without morphologically identifiable retinal inflammation or vessel occlusions based on hematoxylin and eosin stains.

**Table 1:** COVID-19 postmortem donor tissues and SARS-CoV-2 qRT-PCR results.

Type of ocular tissue/ fluid	qRT-PCR for SARS-CoV-2 RNA on right eye	RNA yields (mean, ±SD in ng/μL)	qRT-PCR for SARS-CoV-2 RNA on left eye^a^	RNA yields (mean, ±SD in ng/μL)
Retina	vRNA undetectable	174.7±134.1	vRNA undetectable	136.3±107.1
Vitreous	vRNA undetectable	16.7±30.0	vRNA undetectable	16.3±28.2

SARS-CoV-2: Severe Acute Respiratory Syndrome Coronavirus 2; qRT-PCR: quantitative Reverse Transcriptase-Polymerase Chain Reaction (S-/E-genes, positive/ internal controls); vRNA: viral RNA; SD: Standard deviation.

^a^Received routine procedure of tissue procurement for bio-banking; total no. of COVID-19 postmortem eyes: N = 10.

Thus, no globe had any detectable viral RNA in the vitreous and retina. In addition, histopathological examinations confirmed no morphological sign of damage to retinal vasculature or tissues.

For each set of data analyzed the controls were given. Every positive and negative control was tested valid. Comparing the results between the left and right globes no difference was observed in the detection of SARS-CoV-2 RNA. For each left globe the standard protocol of tissue procurement for corneal banking was employed. The right globes were left naïve.

In this study we did not detect SARS-CoV-2-RNA in human retina and vitreous of COVID-19 cadaveric tissue donors after a severe and protracted course of disease. Albeit not unequivocally positive, the very recent report of a positively tested human retina for SARS-CoV-2 RNA by Casagrande et al. [5] could potentially lead to further adjustments in ophthalmological daily practice and surgery. This study attempted to add knowledge to the topic of a possible ocular reservoir of SARS-CoV-2 in COVID-19 tissue donors in studying human retinal tissue and vitreous samples. Recently the presence of ACE2 and TMPRSS2 was detected in retinal tissues [5]. SARS-CoV-2 might therefore potentially infect the human retina, even though there is no direct proof for the latter. Currently invasive ophthalmological procedures like cataract extraction, intraocular lens implantation, retinal surgery and intravitreal eye injections are taking place on a daily basis with the potential risk of transmitting SARS-CoV-2 via other than the classical respiratory route. The COVID-19 pandemic already necessitates short- and long-term changes to ophthalmological practice. Any reliable study adding information to this topic may impact decisions made in the (near) future in this field.

We described in detail how the tissues were procured and which precautions were taken in order to preclude any contamination with viral RNA during tissue extraction, processing and testing. Moreover, test assay sensitivity was considered as to current standards of practice in microbiology and virology. The employed and reproducible protocols were considered to be effective in preventing SARS-CoV-2 infections during autopsies, tissue retrieval and tissue processing. Employees with the slightest clinical evidence of respiratory symptoms were tested for SARS-CoV-2 using qRT-PCR on pharyngeal swabs. All employees involved remained SARS-CoV-2 negative during the conduct of the study.

Pathological virus-related changes were assessed via histopathological examinations. Recently, a report was published describing pathological, retinal changes in acutely ill COVID-19 patients by Marinho et al. [3]. The acute ischemic nature of the pathological changes might be reversible or might not. As stated by the original group, these retinal lesions appear to be part of an ischemic, microvascular process [4]. In accordance with this, we did not find any persistent pathological changes in individuals succumbed to COVID-19 in this series. Also, one might consider the acute retinal changes in COVID-19 patients described in the literature to be more likely reversible in case of an indirect host-mediated insult. Immune activation may trigger different patho-mechanisms possibly including anti-phospholipid antibodies, complement 5A, reperfusion injury, and immune complexes resulting in tissue injury and/ or vasculopathy as described by Cobo-Soriano et al. [10], Jacob [11], Girn et al. [12], and Nag et al. [13], respectively. Thus, it has to be discussed if these observed retinal changes in acutely affected COVID-19 patients are primary or secondary in nature. If of primary nature one would expect *replicable* viral particles in extracted tissues. To the best of our knowledge no study to date did prove the replicability of SARS-CoV-2 in retinal tissues. A possible route of transmission would be via the blood stream *and* an open or disrupted blood-retina barrier. This seems to be a quite unlikely event in immunocompetent patients, even though described by Chatzouli et al. [14] for herpes simplex virus. As published by Cho et al. [15] a transfusion of blood products obtained from a SARS-CoV-2 infected individual, who had not yet developed signs and symptoms of COVID-19, did not result in disease transmission, although the recipient of the blood product was taking immunosuppressive drugs and was diagnosed with severe aplastic anemia.

As for this and any other study carried out to detect viral RNA in human tissues a number of limitations need to be stated. The procured tissues were representative for a severe and prolonged clinical course of COVID-19 requiring hospitalization and intensive care treatment. Limitation of sample size by following the strict requirements of the ethical board for every case included may affect the ability to generalize the findings. The quality of the data is not affected. The reproducibility of the study design is given. In the interpretation of the test results one needs to consider restricted validity of postmortem testing. The sensitivity of the test was duly considered. As eloquently elaborated by Irene Kuo [16] the experimental setup, proper validation and controls and accurate use of publication descriptors and definitions need to be taken into consideration in interpretation of any study result. As published by Aiello et al. [17] the majority of the available data regarding SARS-CoV-2 colonization of ocular and periocular tissues and secretions have to be considered controversial. The establishment of the virus at its potential portal of entry may as well apply to intraocular tissues and fluids.

Based on our results major follow-up studies would include a larger sample of COVID-19 cadaveric donors. Novel directions may include further testing of nervous tissue of COVID-19 donors including optic nervous tissue. Moriguchi et al. [18] described a case of positive cerebrospinal fluid samples for SARS-CoV-2 in association with clinical signs of an aseptic meningitis/encephalitis. Of interest is the possibility of an indirect damage via SARS-CoV-2 caused by an immune activation as proposed by Song [19]. Although neurological signs such as headache, nausea and vomiting are not specific for a neuro-invasive capability one still needs to consider its possibility as for other coronaviruses as stated by Li et al. [20].

## Conclusions

In the present study no SARS-CoV-2-RNA was detected in human vitreous and retinal samples of deceased COVID-19 patients. In addition, histopathological examinations confirmed no sign of viral damage to retinal vasculature or tissues. SARS-CoV-2 infection of human retinal tissue and/ or vitreous fluid is quite rare—if viral replication possible at all—in COVID-19 postmortem donors. Hence, in our small cohort of COVID-19 deceased patients retinal histpathology appeared normal. Further studies are needed to confirm or refute the results and should also include additional markers to detect immunopathological changes and inflammation in the eye, as also stated by Marinho et al. [4].

## Supporting information

S1 File Laboratory appendix 1. (DOCX)

S2 File Laboratory appendix 2. (DOCX)

S3 File Laboratory appendix 3. (DOCX)

S4 File Human retina and vitreous results. (XLSX)

S5 File Declaration of consent. (DOCX)

S6 File Guidance for submission of COVID-19 postmortem specimens. (DOCX)
