# Supplementary material for: Severe acute respiratory Syndrome-Coronavirus-2: Can it be detected in the retina?
Source: PLoS One. 2021 May 13;16(5):e0251682. doi: 10.1371/journal.pone.0251682 (PMC8118466; doi:10.1371/journal.pone.0251682)
Supplement: S1 File — (DOCX) [file pone.0251682.s001.docx]

**Laboratory appendix 1.** Addition of 600µL RLT (1mL RLT, 10µL ß-mercapto-ethanol RNeasy Kit, QiaCube, QiaSymphony DSP Virus/ Pathogen Kit, Qiagen, Hilden, Germany) and one 5mm-steel ball (Qiagen #69989) to each sample; dissolution in ball mill (Fa. Retsch, 2min, level 100); purification in shredder pillar (Qiagen (#79656), ⭯ centrifugation at 2min at 14.000rpm; addition of same volume of 70% EtOH with DEPC-H2O , non-vortex mix, 700µL for RNeasy spin column, ⭯ centrifugation at 15sec at 14.000rpm, repeat with remaining RLT/EtOH mix); qRT-PCR using RealStar SARS-CoV-2 RT-PCR Kit 1.0 (altona Diagnostics GmbH, Hamburg, Germany) and LightMix® Modular SARS-CoV (COVID19) kit (TIB Molbiol Syntheselabor GmbH, Berlin, Germany).
