# Supplementary material for: Severe acute respiratory Syndrome-Coronavirus-2: Can it be detected in the retina?
Source: PLoS One. 2021 May 13;16(5):e0251682. doi: 10.1371/journal.pone.0251682 (PMC8118466; doi:10.1371/journal.pone.0251682)
Supplement: S2 File — (DOCX) [file pone.0251682.s002.docx]

**Laboratory appendix 2.** Dissolution of lyophilized DNAse in RNAse free water (550µL); addition of RW1 buffer (⭯ centrifugation at 15sec at 14.000rpm, wash column) and of DNAse (10µL) to RDD buffer (70µL), non-vortex mix, centrifugation; addition of DNAse mix to center of column (15min RT); add RW1 buffer (350µL) to column (⭯ centrifugation at 15sec at 14.000rpm), RPE (500µL, ⭯ centrifugation at 15sec at 14.000rpm, wash column); transfer column to 2^nd^ collection tube (⭯ centrifugation at 1min at 14.000rpm) and new Eppendorf tube/ micro-reaction vessel; add RNAse free water (30-50µL, ⭯ centrifugation at 1min at 14.000rpm); second addition of RNAse free water (30-50µL, ⭯ centrifugation at 1min at 14.000rpm) in case of >30µg RNA.
