# Supplementary material for: Severe acute respiratory Syndrome-Coronavirus-2: Can it be detected in the retina?
Source: PLoS One. 2021 May 13;16(5):e0251682. doi: 10.1371/journal.pone.0251682 (PMC8118466; doi:10.1371/journal.pone.0251682)
Supplement: S3 File — (DOCX) [file pone.0251682.s003.docx]

**Laboratory appendix 3.** 10 µl of the RNA, positive or negative control were used for qRT-PCR with the LightMix^®^ SarbecoV E-gene Kit (TIB MOLBIOL, 40-0776-96) in combination with the Roche LightCycler^®^ Multiplex RNA Virus Master kit (Roche, 07083173001). The positive Control was supplied with the LightMix Kit and contained all diagnostic targets (E gene, N gene and RdRP) of SARS and SARS-CoV-2. As negative control the water supplied with the Roche Master kit was used. The reaction mix was prepared as described in the manual.

Data analysis was performed as described in LightCycler II operator’s manual, in brief, colour compensation was selected for multiplex assays and the “Second Derivative Maximum method” was used. The results were shown in 6-carboxyfluorescein (FAM) - channel.

According to the producers’ manual, the sensitivity is 5.2 copies per reaction. A hole genome, synthetic RNA control (Twist Bioscience, #MT007544.1) was also used in qRT-PCR; a consecutive dilution showed, that down to 10 copies per reaction SARS-CoV-2 was detectable (linear correlation) The cut-off was defined as recommended in the LightMix Kit manual: C_P_ value for 10 copies (35.48 ± 0.2) plus 1 cycle and resulted in a C_P_-cut-off value of 36.48.
