# Supplementary material for: Severe acute respiratory Syndrome-Coronavirus-2: Can it be detected in the retina?
Source: PLoS One. 2021 May 13;16(5):e0251682. doi: 10.1371/journal.pone.0251682 (PMC8118466; doi:10.1371/journal.pone.0251682)
Supplement: S5 File — (DOCX) [file pone.0251682.s005.docx]

Attachment 4-7_3_001: Declaration of consent from relatives or public prosecutor's office

I hereby give my consent for the removal of eye tissue (possibly specified _____________________________________________________.)

from my relative

XX, XX ; DD.MM.YYYY

Name, First name; Date of birth

for the purpose of transplantation. This corresponds to the presumed will of the deceased or the will as expressed before demise by the patient herself/ himself.

The relatives consent to the use of the anonymized residual tissue for answering scientific questions: Yes X No X

XX XX; (XX)

Surname and First Name of the relative; (Degree of relationship)

#Number, DD.MM.YYYY, hh:hh

If communicated by telephone: Telephone number, Date, Time

A donation for the purpose of transplantation is agreed (only non-COVID-19 cases).

In the case that, contrary to expectations for non-COVID-19 cases and as expected in COVID-19 cases, a transplantation is not possible, a use for science & research would take place (SARS-CoV-2 and ocular tissue. Examination of donated biomaterial (cornea bank of the department of ophthalmology)).

Procedure and content of the informed consent meeting:

In the event of an unnatural cause of death:

Name of the public prosecutor who gave his consent

If communicated by telephone: Telephone number, Date, Time

Name of informing physician, Signature, Place, Date
