# Supplementary material for: Severe acute respiratory Syndrome-Coronavirus-2: Can it be detected in the retina?
Source: PLoS One. 2021 May 13;16(5):e0251682. doi: 10.1371/journal.pone.0251682 (PMC8118466; doi:10.1371/journal.pone.0251682)
Supplement: S6 File — (DOCX) [file pone.0251682.s006.docx]

Enucleation

The following steps were performed: Final check of the set of instruments; double check identity of postmortem donor and consent form; confirm cause of death (COVID-19); documentation of donor side (right/left), place, and time of enucleation; proper usage of PPE including fitting test of FFP-3/ N-95; inspection of body bag and corpus; preparation of transport media and vessel (left globe: sterile gauze, 10mL NaCl, 10 mL gentamicinsulfate (5mg/mL); right globe: sterile gauze, 10mL NaCl; mark each vessel: COVID-19 donor tissue); flushing of the superior and inferior fornix of the left globe (Betaisodona^®^; 1:10 diluted in sterile NaCl equivalent to 1% of free iodine, flushing with sterile NaCl after 5 min), periocular wiping with Betaisodona^®^; right globe is kept naive; appropriate usage of provided drape, vessel and PPE; performed enucleation with provided single-use surgical set (eyelid blocker, forceps, scissors, hooks) to obtain an intact globe; prosthesis selection, insertion and closure of palpebral fissures; transfer of each globe into specific transport vessel and a re-lockable container marked “COVID-19 donor tissue”; disposal of used PPE and potentially infectious materials.

Preparation

The following steps were performed: Usage of PPE including fitting test of FFP-3/ N-95; disinfection of the sterile workbench (Descosept-AF, desiccation of 15 min); disinfection of globe (left) in diluted iodine solution (5 min in Betaisadona^®^ (7.5 %, Braun, #3864154)/ NaCl (250 ml, Fresenius Kabi, PZN-00809049) 1:20) and thorough rinse (in 50 mL NaCl); keeping right globe naïve; preparation of donor tissues/fluids (surgical set: surgical forceps,15 mm trephine, 30G-cannulas, Kolibri-forceps, Vannas-scissors, Westcott-scissors, hockey knife and vacuum holder) and extraction of fluid-tissue samples for quantitative Reverse Transcription-Polymerase Chain Reaction (qRT-PCR) testing on SARS-CoV-2 RNA (tissue/fluid: retinal tissues and vitreous fluids).
